# Asymmetric c-fos expression in the ventral orbital cortex is associated with impaired reversal learning in a right-sided neuropathy

**DOI:** 10.1186/1744-8069-10-41

**Published:** 2014-06-23

**Authors:** Hugo Leite-Almeida, Marco Rafael Guimarães, João José Cerqueira, Nuno Ribeiro-Costa, Helena Anjos-Martins, Nuno Sousa, Armando Almeida

**Affiliations:** 1 Life and Health Sciences Research Institute (ICVS), School of Health Sciences, University of Minho, Campus de Gualtar, Braga 4710-057, Portugal; 2 ICVS/3B’s - PT Government Associate Laboratory, Braga/Guimarães, Portugal

**Keywords:** Neuropathic pain, Lateralization, Attentional set-shifting task, Reversal learning, Prefrontal cortex, C-fos

## Abstract

**Background:**

Recently we showed that unilateral peripheral neuropathic lesions impacted differentially on rat’s emotional/cognitive behavior depending on its left/right location; importantly, this observation recapitulates clinical reports. The prefrontal cortex (PFC), a brain region morphofunctionally affected in chronic pain conditions, is involved in the modulation of both emotion and executive function and displays functional lateralization. To test whether the PFC is involved in the lateralization bias associated with left/right pain, c-fos expression in medial and orbital areas was analyzed in rats with an unilateral spared nerve injury neuropathy installed in the left or in the right side after performing an attentional set-shifting, a strongly PFC-dependent task.

**Results:**

SNI-R animals required more trials to successfully terminate the reversal steps of the attentional set-shifting task. A generalized increase of c-fos density in medial and orbital PFC (mPFC/OFC), irrespectively of the hemisphere, was observed in both SNI-L and SNI-R. However, individual laterality indexes revealed that contrary to controls and SNI-L, SNI-R animals presented a leftward shift in c-fos density in the ventral OFC (VO). None of these effects were observed in the neighboring primary motor area.

**Conclusions:**

Our results demonstrate that chronic neuropathic pain is associated with a bilateral mPFC and OFC hyperactivation. We hypothesize that the impaired performance of SNI-R animals is associated with a left/right activity inversion in the VO, whose functional integrity is critical for reversal learning.

## Background

Lateralized phenomena in pain have been for many years a topic of heated discussions - confront [1] and [2]. Studies in humans revealed that left-sided pain was more anxiogenic than equivalent right-sided pain (with the similar descriptors of pain e.g. the visual analogue scale) [3]. Also in rats, left-sided neuropathy significantly decreased the time spent exploring the open-arms of the elevated-plus maze (EPM), when compared to right-sided neuropathy or to sham operated animals [4], an indicator of anxiety-like behavior. In addition, in the latter study, it was shown that cognitive behaviors were also affected in a lateralized manner; particularly, a right- but not a left-sided neuropathy was detrimental for working memory, reversal learning (in the context of an attentional set-shifting task, ASST) and inhibitory control [4]. It has been theorized that such phenomena results from: (i.) the organization of the ascending pathways which mainly target the contralateral hemisphere in relation to the sensory/nociceptive input and (ii.) from the functional lateralization of a number of forebrain structures, of notice the prefrontal cortex (PFC; [5]). To test the hypothesis that left- and right-sided pain differentially affect PFC activity, we analyzed c-fos expression in both hemispheres in rats with either a left- or right-sided neuropathy (spared nerve injury model [6], SNI) after the completion of the attentional set-shifting task (ASST). The ASST is an analogue of the human Wisconsin card sorting task, used to test frontal function, in which we have previously demonstrated that right- but not left-sided SNI presented impaired reversal learning [4].

## Results

### Mechanical allodynia

**Figure 1:**
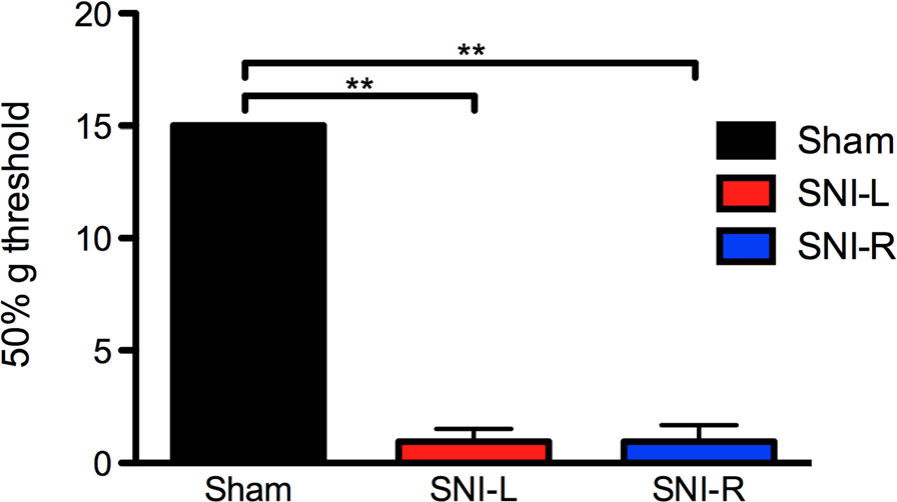
**Mechanical allodynia.** 3-weeks after SNI installation, a significant decrease in the monofilament-induced limb withdrawal threshold was observed independently of the injured side. **P < 0.01. Data presented as mean ± S.E.M.

Using the up-and-down assay to assess the 50% withdraw threshold we firstly showed that 3 weeks after the installation of the SNI model, there were marked differences to Von Frey monofilament probing between the three experimental groups (F_2,21_ = 1518.301; P < 0.001). Post hoc analyses revealed a statistically significant threshold drop in the SNI groups (P < 0.001 in both sham *vs* SNI-L and sham *vs* SNI-R comparisons). No differences were however found between the two neuropathic groups SNI-L and SNI-R (Figure 1).

### Attentional set-shifting task

**Figure 2:**
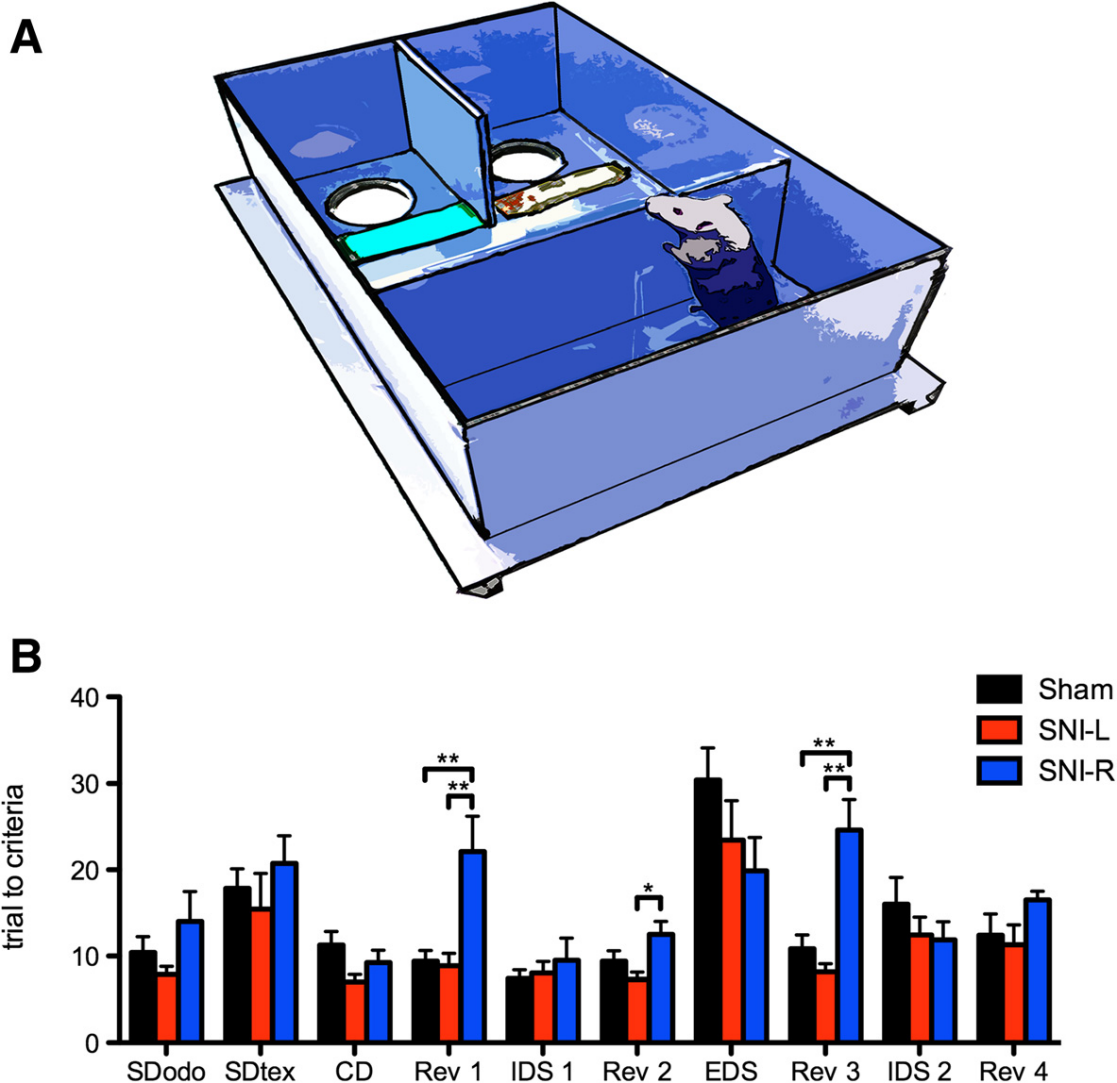
**Attentional set-shifting task. (A)** The ASST was performed in a custom-built rectangular 70 x 40 x 20 cm (L, W, H) arena. **(B)** SNI-R animals required a significantly higher number of trials to criteria than sham and SNI-L animals to accomplish reversals (Rev) 1, 2 and 3 of the ASST task. No differences were found between groups in the other ASST steps. **P < 0.001. Data presented as mean ± S.E.M.

No differences were observed in the simple discrimination tasks for odor (SD_odo_; F_2,21_ = 1.589, n.s.) and texture (SD_tex_; F_2,21_ = 0.674, n.s.) indicating that all groups are able to equally discriminate both perceptual dimensions (Figure 2B). In the ASST proper, however, a group effect was observed in the reversal learning tasks Rev 1–3 (F_2,21_ = 7.561, P < 0.01; F_2,21_ = 4.611, P < 0.05; F_2,21_ = 13.470; P < 0.001, respectively). In these cases, SNI-R required more trials to accomplish the criteria than sham or SNI-L groups as revealed by post hoc analyses (Figure 2B). In Rev 4, the same tendency was observed, although it did not reach statistical significance (F_2,21_ = 2.045; n.s.). In all other steps of the ASST, compound discrimination, intradimensional shift (IDS1-2) and extradimensional shift (EDS) no significant differences were detected (CD, F_2,21_ = 2.475; IDS1, F_2,21_ = 0.337; EDS, F_2,21_ = 1.774; IDS2, F_2,21_ = 0.821) (Figure 2B).

### c-fos^+^ cell density in medial and orbital prefrontal cortices

**Figure 3:**
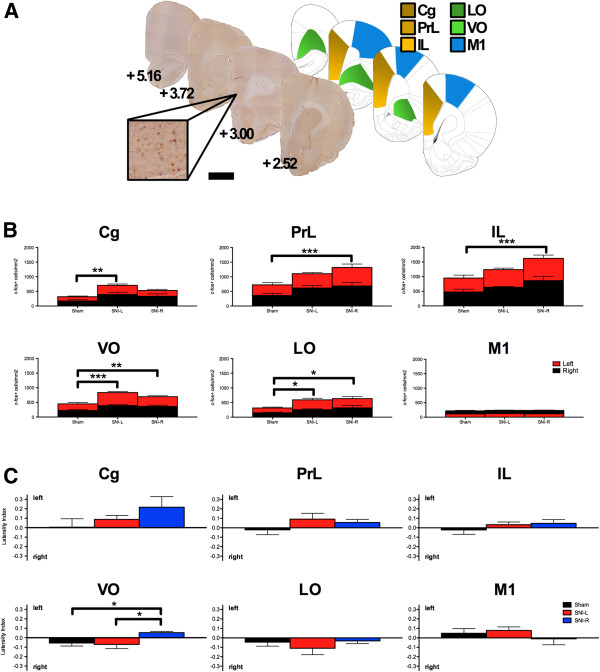
**c-fos expression in the prefrontal cortex. (A)** Exemplificative c-fos stained sections, the respective Paxinos and Watson atlas diagrams [7] and respective distance to Bregma (mm) are given. Scale bar, 2 mm (100 μm inset). **(B)** SNI groups present a higher c-fos density when compared to sham controls in the mPFC and OFC but not in the control area M1. The hemisphere side was not a determining factor for the differences between the groups. **(C)** SNI-R contrary to sham/SNI-L presented a leftward shift in the laterality index in the VO following ASST completion. The functional integrity of this area is critical to Rev execution. *P < 0.05, **P < 0.01 and ***P < 0.001. Data presented as mean ± S.E.M.

c-fos^+^ cell densities were analyzed in medial and orbital prefrontal (mPFC and OFC, respectively) regions from both hemispheres and expressed as c-fos^+^/mm^2^ (Figure 3B). Statistically significant differences were observed between the three groups in the mPFC areas cingulate (Cg) (F_2,43_ = 5.446; P < 0.01), prelimbic (PrL) (F_2,43_ = 5.314; P < 0.01) and infralimbic (IL) (F_2,43_ = 5.657; P < 0.01) and in the OFC areas ventral orbital (VO) (F_2,43_ = 11.640; P < 0.001) and lateral orbital (LO) (F_2,43_ = 5.059; P < 0.05). In all cases, the differences resulted from a generalized increase in c-fos expression in the SNI groups (Figure 3B). Post hoc tests revealed statistically significant differences in c-fos^+^ cells density between the sham and SNI-L animals in the Cg cortex (P = 0.006) but not between sham and SNI-R (P = 0.160). In the ventral mPFC regions the inverse was observed, i.e., statistical significant differences were present between sham and SNI-R groups (PrL and IL, P < 0.001) but not in sham/SNI-L comparisons (PrL, P = 0.122; IL, P = 0.357). In the OFC, however, post hoc analyses revealed significant differences between the sham operated group and both SNI groups (VO, P_sham/SNI-L_ < 0.001 and P_sham/SNI-R_ = 0.01; LO, P_sham/SNI-L_ and P_sham/SNI-R_ < 0.05). Similar analyses in the primary motor cortex (M1) failed to reveal any difference between the experimental groups (F_2,43_ = 0.178; N.S.). In any of the areas analyzed, neither the hemisphere side nor the interaction between the experimental group and side had a statistically significant effect. Within group comparisons of c-fos^+^ cell densities between left and right hemispheres also failed to reveal differences in any of the mPFC, OFC areas and the control M1 cortex.

To further detail the interhemispheric expression of c-fos within each experimental subject after ASST, we calculated and compared the laterality index (Figure 3C). Comparisons between the three experimental groups revealed a statistical significant effect in the VO (F_2,21_ = 4.892; P = 0.019) but not in any other area analyzed. Opposing lateralization biases between sham/SNI-L and SNI-R to the right and left hemispheres, respectively, accounted for much of this effect. Additionally, the analyses of M1 laterality index also provided no indication of lateralized effects (F_2,21_ = 0.754; N.S.).

## Discussion

The PFC presents robust morphological and functional alterations in chronic pain conditions both in human brain imaging studies [8] and in the rat [9]. Not surprisingly, PFC-mediated functions - working memory, impulse inhibition, attention and behavioral flexibility – are impaired in these patients and experimental models [10]. In a recent study, we reported that the side of the neuropathic lesion critically influences the outcome of the behavioral impairments [4] (such lateralization bias had also been observed in human subjects [3]).

In the present study, we observed a bilateral increase in c-fos density in both SNI groups therefore not providing an anatomofunctional explanation for the sidedness effect observed in the Rev - i.e. SNI-R but not SNI-L present an impaired ability to preform this task. This finding most probably reflects a hyperexcitated state of the PFC of SNI animals, which has already been described in chronic pain conditions [11]. Indeed, in patients with lateralized painful neuropathy a generalized increase (and mostly bilateral) of rCBF is observed in a number of frontal areas including the insula, prefrontal and cingulate cortices [12]. Similarly, ^14^C-2-deoxyglucose uptake – reflecting energetic demand/activity – was increased in a rat neuropathy model in the same regions [13]. Again, the trend was mostly bilateral.

However, it has been previously shown that the central effects of unilateral peripheral neuropathy maintain to a certain extent, a lateralized character. These include alterations in synaptic proteins [14] or pro-inflammatory cytokines (particularly IL-1β) [15-17]. Indeed, the rightward c-fos lateralization bias observed in the VO of sham operated control animals performing the ASST was disrupted by the presence of a unilateral peripheral neuropathic lesion in the right but not in the left sciatic nerve. Rev and extradimensional shift EDS ability depend on the functional integrity of the OFC and ventral mPFC, respectively, as demonstrated in monkeys [18], rats [19] and mice [20] lesion studies. This anatomical specificity had also been observed in the patterns of c-fos expression after Rev or EDS [21] suggesting that this abnormal lateralization bias might be associated with the impaired Rev ability of SNI-R.

We demonstrate that chronic neuropathic pain is associated with an increased c-fos expression in the mPFC and OFC but not in the M1. In SNI-L animals this effect is particularly strong in the Cg while in SNI-R animals it is shifted toward more ventral mPFC areas, IL and PrL. It is tempting to speculate on a putative association between this pattern and the differential emotional/cognitive impairments observed in SNI-L and SNI-R rats [4]. Even though we cannot entirely isolate pain- and task-related activity, the inversion of the side bias observed in the VO of SNI-R in comparison with the pain-free group, is highly suggestive a of causal relation with the behavioral impairments observed in the former.

## Materials and methods

### Experimental subjects

3 months old, male Wistar Han rats (Charles River Laboratories, Barcelona, Spain) were used in all experiments. Food availability was restricted to 1 h/day (7:00–8:00 p.m.) during the time frame of the attentional set-shifting task (ASST) training and execution; animals’ weight was controlled to ensure that it was maintained above 10-15% of the initial weight. All procedures with animals were performed according to the guidelines of European Communities Council Directive 2010/63/EU.

### Neuropathy model and pain assessment

The SNI model of peripheral neuropathy was used in this study [6]. The model was installed in the left (SNI-L; N = 7) or right (SNI-R; N = 8) hindpaw following previously described procedures [4]. A group of left/right sham-operated animals was also included (N = 7). At the third week post surgery, mechanical allodynia was assessed using the up-and-down method [22].

### Attentional-set shifting task

At the 4^th^ week post surgery the ASST was initiated. The paradigm was performed in a rectangular arena. In the first third a wall separated two compartments each containing a bowl with sawdust. In the beginning of each trial, the wall separating the last third of the arena was lifted, allowing the animals to reach the bowls (Figure 2A). A successful trial was considered if the animal dug the correct bowl and retrieved the reward (cheerio cereal). In the compound discrimination (CD) tasks, 2 odors (aromatic oils rubbed in the bowls’ rim) and 2 textures (placed close to the bowls) were simultaneously present. In the first CD task, odor was the relevant cue; this was followed by its reversal (Rev 1) i.e. the previously irrelevant odor became the signal for reward. A new pair of odors and textures was then introduced maintaining odor as the relevant dimension (intradimensional shift; IDS1); Rev 2 followed IDS1. The relevant dimension was then shifted from odor to texture (extradimensional shift; EDS) followed by Rev 3, IDS 2 and Rev 4. Tasks were terminated when animals preformed 6 successful trials consecutively. The organization of the trials is given in Table 1.

**Table 1:** Attentional set-shifting task trial organization and sequence

**triaI**	**1**		**2**		**3**		**4**		**5**		**6**		**7**		**8**		**9**		**10**	
**bowl**	**L**	**R**	**L**	**R**	**L**	**R**	**L**	**R**	**L**	**R**	**L**	**R**	**L**	**R**	**L**	**R**	**L**	**R**	**L**	**R**
S D_0d0_	O_2_	**O**_**1**_	**O**_**1**_	O_2_	**O**_**1**_	O_2_	O_2_	**O**_**1**_	**O**_**1**_	O_2_	O_2_	**O**_**1**_	O_2_	**O**_**1**_	O_2_	**O**_**1**_	**O**_**1**_	O_2_	**O**_**1**_	O_2_
SD_tex_	T_2_	**T**_**1**_	**T**_**1**_	T_2_	**T**_**1**_	T_2_	T_2_	**T**_**1**_	**T**_**1**_	T_2_	**T**_**1**_	T_2_	**T**_**1**_	T_2_	**T**_**1**_	T_2_	T_2_	**T**_**1**_	T_2_	**T**_**1**_
CD	**O**_**3**_	O_4_	O_4_	**O**_**3**_	O_4_	**O**_**3**_	O_4_	**O**_**3**_	**O**_**3**_	O_4_	**O**_**3**_	O_4_	O_4_	**O**_**3**_	O_4_	**O**_**3**_	**O**_**3**_	O_4_	**O**_**3**_	O_4_
	T_3_	T_4_	T_3_	T_4_	T_4_	T_3_	T_4_	T_3_	T_4_	T_3_	T_3_	T_4_	T_3_	T_4_	T_3_	T_4_	T_3_	T_4_	T_4_	T_3_
Rev_1_	O_3_	**O**_**4**_	O_3_	**O**_**4**_	O_3_	**O**_**4**_	**O**_**4**_	O_3_	**O**_**4**_	O_3_	O_3_	**O**_**4**_	**O**_**4**_	O_3_	**O**_**4**_	O_3_	O_3_	**O**_**4**_	**O**_**4**_	O_3_
	T_4_	T_3_	T_4_	T_3_	T_4_	T_3_	T_4_	T_3_	T_3_	T_4_	T_3_	T_4_	T_3_	T_4_	T_4_	T_3_	T_3_	T_4_	T_3_	T_4_
IDS_1_	**O**_**5**_	O_6_	**O**_**5**_	O_6_	**O**_**5**_	O_6_	O_6_	**O**_**5**_	O_6_	**O**_**5**_	O_6_	**O**_**5**_	O_6_	**O**_**5**_	O_6_	**O**_**5**_	**O**_**5**_	O_6_	**O**_**5**_	O_6_
	T_5_	T_6_	T_5_	T_6_	T_6_	T_5_	T_5_	T_6_	T_6_	T_5_	T_5_	T_6_	T_5_	T_6_	T_6_	T_5_	T_6_	T_5_	T_6_	T_5_
Rev_2_	**O**_**6**_	O_5_	O_5_	**O**_**6**_	O_5_	**O**_**6**_	O_5_	**O**_**6**_	**O**_**6**_	O_5_	**O**_**6**_	O_5_	O_5_	**O**_**6**_	**O**_**6**_	O_5_	**O**_**6**_	O_5_	O_5_	**O**_**6**_
	T_5_	T_6_	T_6_	T_5_	T_6_	T_5_	T_5_	T_6_	T_6_	T_5_	T_5_	T_6_	T_5_	T_6_	T_6_	T_5_	T_6_	T_5_	T_6_	T_5_
EDS	O_7_	O_8_	O_8_	O_7_	O_7_	O_8_	O_7_	O_8_	O_7_	O_8_	O_8_	O_7_	O_7_	O_8_	O_8_	O_7_	O_8_	O_7_	O_8T7_	O_7_
	**T**_**7**_	T_8_	T_8_	**T**_**7**_	**T**_**7**_	T_8_	**T**_**7**_	T_8_	T_8_	**T**_**7**_	T_8_	**T**_**7**_	T_8_	**T**_**7**_	T_8_	**T**_**7**_	**T**_**7**_	T_8_	**T**_**7**_	T_8_
Rev_3_	O_8_	O_7_	O_8_	O_7_	O_7_	O_8_	O_7_	O_8_	O_7_	O_8_	O_8_	O_7_	O_8_	O_7_	O_8_	O_7_	O_7_	O_8_	O_7_	O_8_
	T_7_	**T**_**8**_	T_7_	**T**_**8**_	T_7_	**T**_**8**_	**T**_**8**_	T_7_	T_7_	**T**_**8**_	**T**_**8**_	T_7_	**T**_**8**_	T_7_	**T**_**8**_	T_7_	T_7_	**T**_**8**_	**T**_**8**_	T_7_
IDS_2_	O_9_	O_10_	O_9_	O_10_	O_10_	O_9_	O_9_	O_10_	O_9_	O_10_	O_10_	O_9_	O_10_	O_9_	O_9_	O_10_	O_10_	O_9_	O_10_	O_9_
	**T**_**9**_	T_10_	T_10_	**T**_**9**_	**T**_**9**_	T_10_	**T**_**9**_	T_10_	T_10_	**T**_**9**_	T_10_	**T**_**9**_	T_10_	**T**_**9**_	T_10_	**T**_**9**_	**T**_**9**_	T_10_	**T**_**9**_	T_10_
Rev_4_	O_9_	O_10_	O_10_	O_9_	O_9_	O_10_	O_9_	O_10_	O_9_	O_10_	O_10_	O_9_	O_10_	O_9_	O_9_	O_10_	O_9_	O_10_	O_10_	O_9_
	T_9_	**T**_**10**_	T_9_	**T**_**10**_	T_9_	**T**_**10**_	**T**_**10**_	T_9_	T_9_	**T**_**10**_	T_9_	**T**_**10**_	**T**_**10**_	T_9_	**T**_**10**_	T_9_	T_9_	**T**_**10**_	**T**_**10**_	T_9_

Odors and textures are presented separately (SD_odo_, SD_tex_) or combined (CD, Rev 1–4, IDS ½, EDS) in simple and compound discrimination steps, respectively. If at the 10^th^ trial the learning criteria were not successfully achieved, the sequence was restarted. Relevant dimension on each trial are bold labeled. Odors: O_1_-strawberry; O_2_-camellia; O_3_-lemon; O_4_-papaya; O_5_-rose; O_6_-vanilla; O_7_-lotus; O_8_-cinnamon; O_9_-mango; O_10_-eucalyptus; Textures: T_1_-styrofoam; T_2_-sponge; T_3_-sandpaper; T_4_-scourer; T_5_-sponge cloth 1; T_6_- sponge cloth 2; T_7_-cardboard; T_8_- velvet; T_9_-carpet; T_10_-foam. L-left compartment; R-right compartment.

### Tissue collection and c-fos immunohistochemistry

90 minutes after ASST termination animals were deeply anaesthetized and perfused intracardially with PBS followed by 4% paraformaldehyde. Brains were then removed and 50 μm coronal sections were obtained for immunoreaction. Brain sections were then incubated (overnight; RT) with a 1:2000 anti-c-fos serum (PC38 Rabbit; Calbiochem®, Nottingham, UK). After incubation with a 1:200 biotinylated secondary antibody (polyclonal swine anti-rabbit; E0353, Dako, Glostrup, Denmark), immunoreactivity was visualized using a chromogen reaction (Vector Laboratories, Burlingame, USA).

### Cell counting procedures

c-fos^+^ cells were counted using Visiopharm Integrator System software (version 2.123.0; Hoersholm, Denmark) and a motorized microscope (BX-51; Olympus) connected to a digital camera (U-TV1X-2; Olympus). Square probes (2500 μm^2^) were placed over the selected areas, evenly spaced, covering 20% of the total defined area. The random positioning of this grid by the software ensured an unbiased and effective sampling. Spheroid/ovoid brownish nuclei within the probes were considered to be c-fos^+^ and were counted. The experimenter performing the sampling was blind to the experimental condition of the subjects. The interhemispheric expression of c-fos was calculated by the laterality index defined as

$${\text{Laterality}}_{\text{index}}\;=\;\frac{{\text{density}}_{\mathrm{left}}\;-\;{\text{density}}_{\text{right}}}{{\text{density}}_{\text{left}}\;+\;{\text{density}}_{\text{right}}}$$

### Statistics

Data is presented as mean ± S.E.M. and analyzed using one- or two-way analysis of variance (1-w- or 2-w-ANOVA) followed by Tukey’s post-hoc test for multiple comparisons (comparison of three or more groups), or t-test (comparison of two groups). P < 0.05 was considered to represent a significant difference.

## Abbreviations

ASST: Attentional set-shifting task; CD: Compound discrimination; Cg: Cingulate cortex; EDS: Extradimensional shift; IDS: Intradimensional shift; IL: Infralimbic cortex; LO: Lateral orbital cortex; mPFC: Medial prefrontal cortex; OFC: Orbital frontal cortex; PFC: Prefrontal cortex; PrL: Prelimbic cortex; rCBF: Regional cerebral blood flow; Rev: Reversal; SD_odo/tex_: Simple discrimination for odor/texture; SNI: Spared nerve injury; SNI-L(R): Left-(right-) sided SNI; VO: Ventral orbital cortex.

## Competing interests

The authors declare taht they have no competing interests.

## Authors’ contributions

HLA, JJC, NS and AA designed the study; HLA, MRG, NRC, HAM and JJC performed the experiments; HLA, MRG and JJC analyzed the data; HLA, JJC, NS and AA wrote the manuscript. All authors read and approved the final manuscript.
